# Supplementary material for: Liquid biopsy for the diagnosis of EBV-positive Burkitt’s lymphoma in endemic areas
Source: Nat Med. 2026 Mar 19;32(5):1754–62. doi: 10.1038/s41591-026-04291-z (PMC13190244; doi:10.1038/s41591-026-04291-z)
Supplement: Supplementary file 1 — Supplementary Figs. 1–3 and Tables 1–6. [file 41591_2026_4291_MOESM1_ESM.pdf]

nature medicine

Supplementary information https://doi.org/10.1038/s41591-026-04291-z

Liquid biopsy for the diagnosis of EBV-positive Burkitt's lymphoma in endemic areas

In the format provided by the authors and unedited

SUPPLEMENTARY DATA

Supplementary Figures

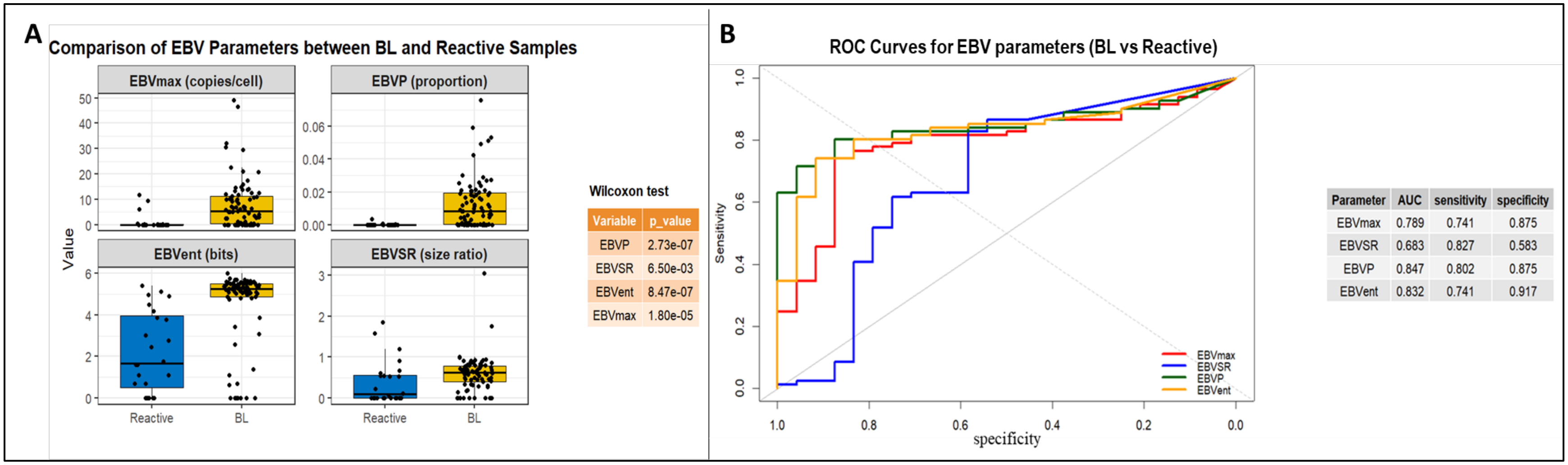

Supplementary Data Figure 1 | Comparison of Epstein–Barr virus (EBV) biomarkers between Burkitt lymphoma and reactive samples.

A, box-and-jitter plots showing distributions of EBVmax (copies per cell), EBVP (proportion), EBVent (entropy) (bits) and EBVSR (size ratio) in reactive and Burkitt lymphoma (BL) samples. Centre lines denote medians; boxes indicate interquartile ranges; whiskers extend to 1.5×IQR; points represent individual samples. Group comparisons were performed using two-sided Wilcoxon rank-sum tests. **B,** receiver operating characteristic (ROC) curves evaluating the diagnostic performance of individual EBV parameters for discriminating BL from reactive samples. The table summarises area under the curve (AUC), sensitivity and specificity for each marker.

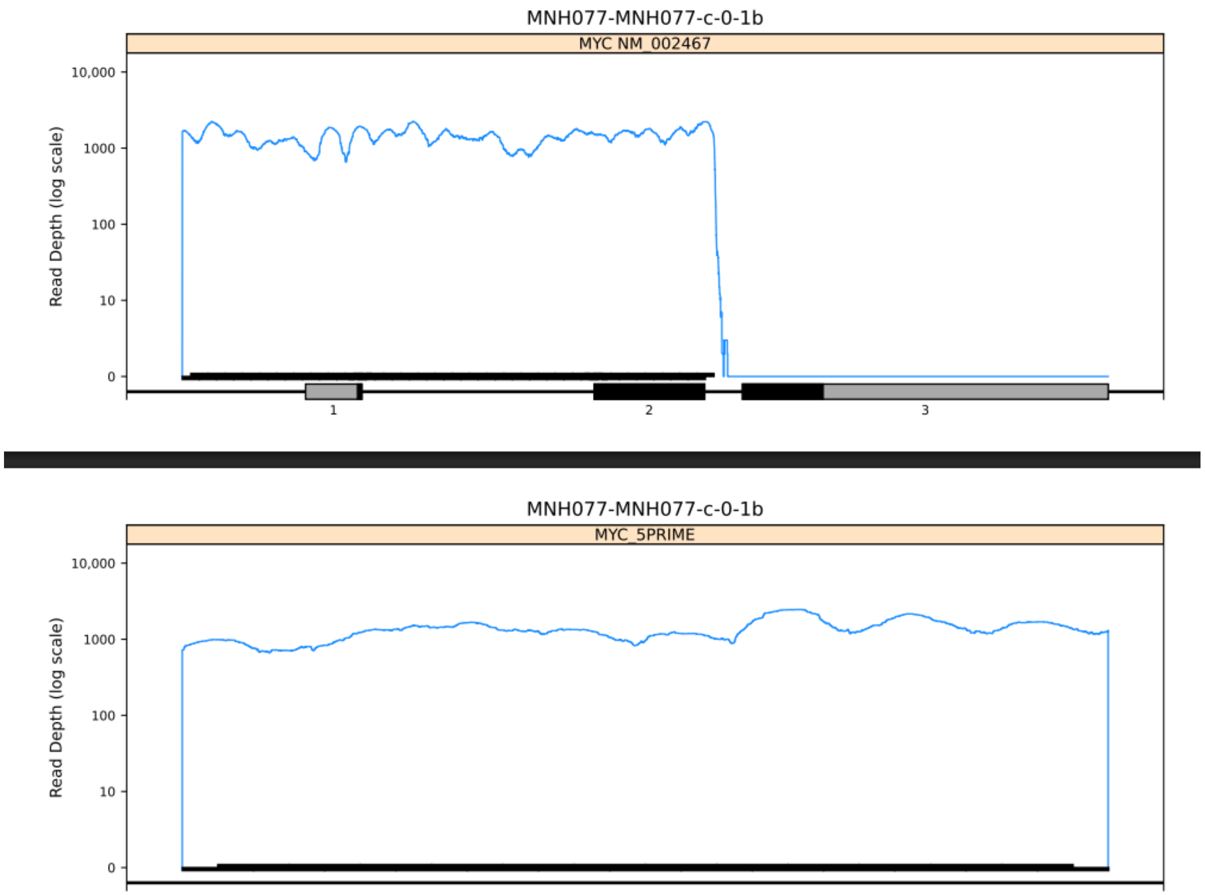

Supplementary Data Figure 2 | Coverage of the MYC gene assessed by targeted sequencing. CoverMi plots showing sequencing coverage across the MYC gene. The x-axis represents genomic position along MYC, with exons 1–3 indicated. Black bars denote regions targeted by the sequencing panel. The y-axis shows read depth on a logarithmic scale.

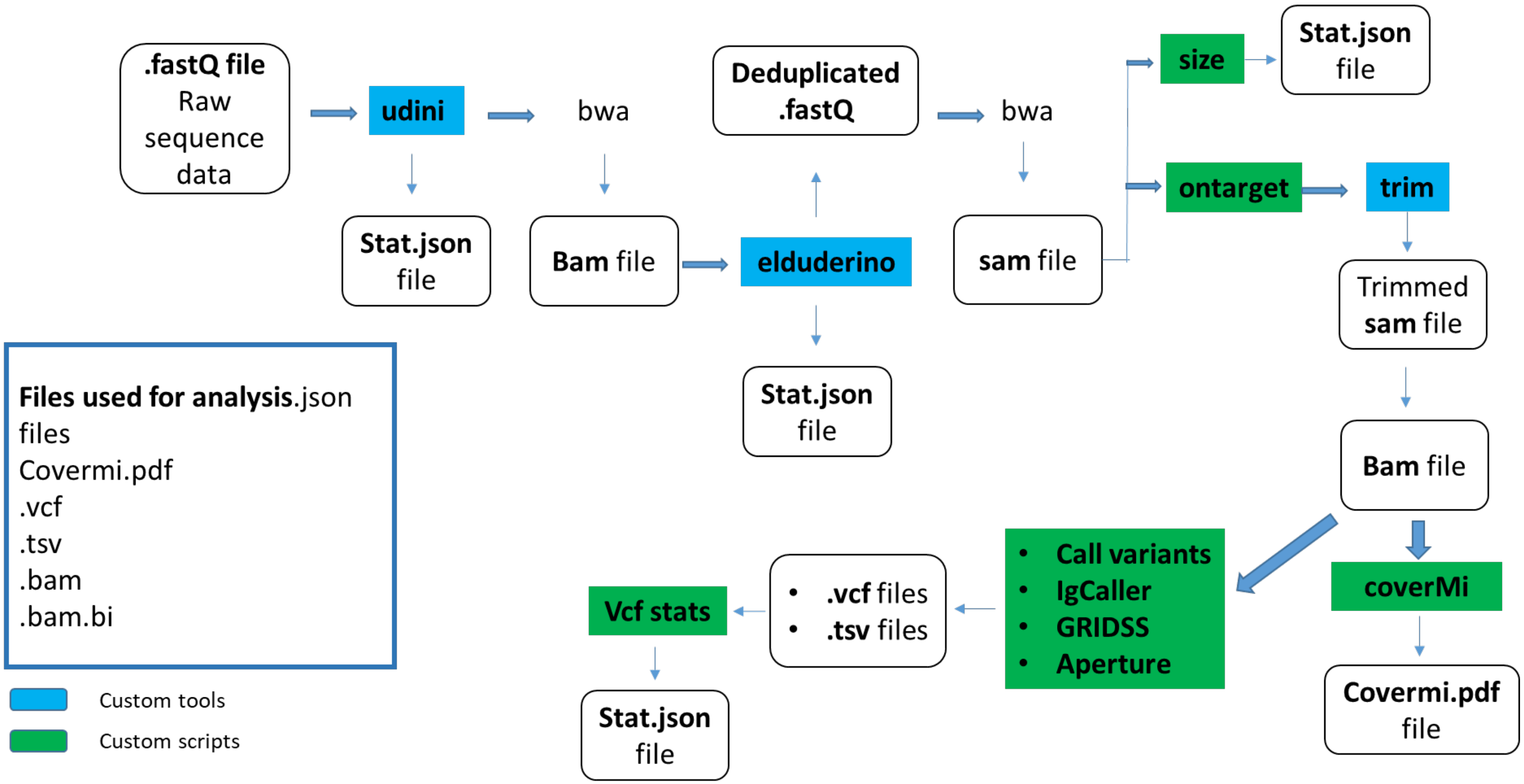

Supplementary Data Figure 3 | Bioinformatic pipeline for cfDNA sequencing data analysis. Schematic overview of the analysis workflow from raw sequencing data to variant calling and coverage assessment. Raw FASTQ files are processed for deduplication and alignment, generating BAM and SAM files with associated quality metrics (Stat.json). Target size and on-target read trimming are applied prior to variant calling using IgCaller, GRIDSS and Aperture, producing VCF and TSV outputs.

Coverage across targeted regions is assessed using CoverMi, generating coverage reports (CoverMi.pdf). All intermediate and final outputs used for downstream analyses are indicated.

Supplementary Tables

Table 1: Full clinical and liquid biopsy characteristics of Phase I participants stratified by BL and non-BL diagnosis (N = 212 children and young adults with clinically suspected lymphoma)

Characteristic	Overall[1]	BL[1]	Non-BL[1]	p-value[2]	FDR p-value[3]	Test	Effect Size[4]
Age (years)	10.3 (6.8, 14.1)	9.9 (6.8, 12.5)	10.7 (6.9, 15.7)	0.0429	0.0480	Wilcoxon	0.139
Sex				0.2949	0.2949	Chi-squared	0.062
Female	72 (34%)	24 (30%)	48 (37%)				
Male	140 (66%)	57 (70%)	83 (63%)				
Duration of symptoms (months)	3 (1, 6)	2 (1, 3)	4 (2, 10)	1.90e-09	4.51e-09	Wilcoxon	0.413
Tumor site				5.43e-16	5.16e-15	Chi-squared	0.547
Jaw/Abdominal	103 (49%)	68 (84%)	35 (27%)				
Other	109 (51%)	13 (16%)	96 (73%)				
Lactate Dehydrogenase (IU/L)	656 (385, 1,312)	945 (553, 1,633)	516 (343, 824)	7.50e-06	1.10e-05	Wilcoxon	0.348
Median VAF	0.01 (0.00, 0.06)	0.07 (0.02, 0.13)	0.02 (0.00, 0.03)	3.76e-15	1.78e-14	Wilcoxon	0.540
ctDNA (HGE/ml)	153 (0, 664)	530 (166, 1,579)	28 (0, 241)	9.93e-12	1.89e-11	Wilcoxon	0.468
cfDNA (HGE/ml)	7,995 (4,242, 20,614)	9,500 (5,446, 22,273)	7,339 (3,608, 18,656)	0.0863	0.0910	Wilcoxon	0.118
MYC intron 1 mutation count	1 (0, 13)	15 (6, 28)	0 (0, 1)	2.03e-19	3.85e-18	Wilcoxon	0.619

Characteristic	Overall[1]	BL[1]	Non-BL[1]	p-value[2]	FDR p-value[3]	Test	Effect Size[4]
MYC exon 2 mutation count	0.00 (0.00, 0.50)	0.00 (0.00, 2.00)	0.00 (0.00, 0.00)	1.07e-07	1.56e-07	Wilcoxon	0.365
EBER1 (copies/cell)	0.1 (0.0, 3.1)	3.1 (0.1, 6.6)	0.0 (0.0, 0.3)	2.00e-12	6.64e-12	Wilcoxon	0.483
EBER2 (copies/cell)	0.07 (0.00, 2.47)	3.06 (0.15, 5.51)	0.00 (0.00, 0.22)	2.10e-12	6.64e-12	Wilcoxon	0.483
EBNA2 (copies/cell)	0.0 (0.0, 2.6)	1.2 (0.0, 10.1)	0.0 (0.0, 0.2)	2.41e-06	3.82e-06	Wilcoxon	0.324
EBVmax (copies/cell)	0.1 (0.0, 5.2)	5.3 (0.4, 11.3)	0.0 (0.0, 0.4)	3.07e-12	8.34e-12	Wilcoxon	0.479
EBV fragment size ratio	0.43 (0.00, 0.67)	0.63 (0.40, 0.78)	0.15 (0.00, 0.55)	7.97e-08	1.51e-07	Wilcoxon	0.369
EBV proportion	0.000 (0.000, 0.008)	0.008 (0.000, 0.019)	0.000 (0.000, 0.001)	4.71e-13	2.24e-12	Wilcoxon	0.497
EBV fragment entropy, bits	4.64 (1.10, 5.22)	5.25 (4.88, 5.49)	2.43 (0.00, 4.90)	1.05e-13	6.63e-13	Wilcoxon	0.511
Autosomal fragment entropy, bits	6.81 (6.66, 6.95)	6.91 (6.75, 7.04)	6.76 (6.60, 6.91)	1.15e-04	1.45e-04	Wilcoxon	0.265
MYC-IG Translocation	39 (18%)	39 (48%)	0 (0%)	1.47e-18	2.80e-17	Chi-squared	0.591

[1]Median (Q1, Q3) for continuous; n (%) for categorical

[2]Exact p-values from Wilcoxon rank-sum or Chi-squared tests

[3]FDR-adjusted p-values (Benjamini-Hochberg)

[4]r for Wilcoxon; Cramér's V for Chi-squared

Table 2: False-negative vs true-positive BL cases based on the comprehensive diagnostic model

Characteristic	False Negative N = 11[1]	True Positive N = 70[1]	p-value[2]
**Age**	12.2 (6.3, 18.0)	9.6 (6.8, 12.1)	0.2
**Gender**	7 / 11 (64%)	50 / 70 (71%)	0.7
**Duration of symptoms (months)**	3.63 (1.13, 4.77)	1.73 (1.00, 2.80)	0.074
**Tumor site (jaw/abdomen)**	4 / 11 (36%)	64 / 70 (91%)	**<0.001**
**LDH**	869 (403, 979)	924 (536, 1,733)	0.3

[1]Median (Q1, Q3); n / N (%)

[2]Wilcoxon rank sum test; Fisher's exact test

Table 3: Sequencing metrics stratified by Presence of Translocation in BL cases, N = 81

Characteristic	Overall, N = 81[1]	Translocation Absent N = 42[1]	Translocation Present N = 39[1]	p-value[2]
**Coverage (%)**	99.00 (98.00, 99.00)	99.00 (98.00, 99.00)	99.00 (98.00, 99.00)	0.7
**ti_tv_ratio**	1.45 (1.29, 1.53)	1.47 (1.35, 1.57)	1.38 (1.28, 1.49)	0.053
**off_target**	60 (55, 66)	61 (55, 67)	59 (56, 64)	0.2
**duplicates**	40 (29, 60)	44 (28, 62)	39 (32, 58)	0.7
**seq_yield**	5.79 (4.79, 6.05)	5.72 (4.75, 6.00)	6.00 (4.85, 6.10)	0.10

**seq_align**	2.96 (2.13, 4.11)	3.27 (2.13, 4.15)	2.89 (1.94, 3.78)	0.4
**seq_error**	0.51 (0.43, 0.64)	0.51 (0.44, 0.76)	0.50 (0.42, 0.60)	0.2
**seq_cluster**	89 (84, 92)	89 (79, 92)	91 (86, 93)	0.2
**seq_q30**	95.0 (90.7, 96.1)	94.0 (89.6, 95.6)	95.4 (92.1, 96.1)	0.2
**Median Fragment Size**	166.0 (164.0, 169.0)	167.0 (163.0, 169.0)	166.0 (164.0, 171.0)	0.7
**Mean Depth**	2,006 (1,551, 2,818)	1,814 (1,162, 2,315)	2,287 (1,746, 3,118)	0.008
**Sequencing Error Rate**	0.010 (0.005, 0.017)	0.010 (0.007, 0.017)	0.010 (0.005, 0.015)	0.5
**Mean depth of MYC**	1,586 (1,156, 2,234)	1,481 (960, 1,932)	1,866 (1,349, 2,753)	0.004
**Mean depth of MYC_5PRIME**	1,630 (1,148, 2,323)	1,454 (933, 1,836)	1,873 (1,297, 2,782)	0.004

[1] Median (Q1, Q3)

[2] Wilcoxon rank sum test

Table 4: Genomic Targets for EBVL Next Generation Sequencing Panel

Genome	*Reference*	*Ref.*	*Transcript*	*Target (exons)*	*Chr*	*Start*	*Stop*	*Comments*
EBV	EBV-WT	EBER1	NC_007605.1			6,629	6,795	100% identical to EBV type 2 EBER1, for EBV detection
EBV	EBV-WT	EBER2	NC_007605.1			6,956	7,128	100% identical to EBV type 2 EBER2, for EBV detection

EBV	EBV-WT	EBNA2	YP_401644.1_10			659	790	Specific to EBV Type 1. For EBV strain identification
Human	hg19	5' MYC			8	128,746,246	128,747,679	Class 2 breakpoints, covers 20 breakpoints from study referenced
Human	hg19	MYC	NM_002467.5	1	8	128,747,680	128,748,869	Class 1/2 breakpoints, typically seen in eBL
Human	hg19	MYC	NM_002467.5		8	128,748,870	128,750,493	Class 1 breakpoints
Human	hg19	MYC	NM_002467.5	2	8	128,750,494	128,751,265	Commonly mutated in BL. Included as 'safety net' encase translocations aren't captured
Human	hg19	ITPKB	NM_002221.3	2	1	226,923,228	226,925,159	
Human	hg19	XPO1	NM_003400	1-15	2	61,719,470	61,719,471	c.1711-12 (p.E517) cHL hotspot, but also PMBL (1711) & CLL (1712)
Human	hg19	TNFAIP3	NM_001270508.1	1-9	6	138,188,581	138,204,449	
Human	hg19	NFKBIE	NM_004556.2	1-6	6	44,226,953	44,233,502	
Human	hg19	STAT6	NM_001178078	11-18	12	57,492,575	57,498,369	c.1249A>T and c.1255G>A hotspots, but common in PMBL
Human	hg19	EP300	NM_001429.3	24-29	22	41,564,453	41,569,788	
Human	hg19	BTK	NM_001287344	1-19	X	100,604,438	100,641,183	

Human	hg19	B2M	NM_004048.2	1-2	15	45,003,675	45,007,899	Mutations frequent in splice donor/acceptor region of intron 1
Human	hg19	SOCS1	NM_003745	1-2	16	11,348,262	11,350,036	
Human	hg19	CSF2RB	NM_00395.3	6, 14	22	37,325,681	37,335,000	
Human	hg19	TP53	NM_000546.5	4-8	17	7,577,019	7,579,590	BL and HL
Human	hg19	ID3	NM_002167.4	1-3	1	23,884,409	23,886,285	
Human	hg19	IGHA2	ENST00000390539.2	1-3	14	106,053,226	106,054,732	IG C gene
Human	hg19	IGHA2			14	106,056,222	106,056,838	Sα region
Human	hg19	IGHA2			14	106,057,056	106,057,601	Sα region
Human	hg19	IGHA1	ENST00000390547.2	1-3	14	106,173,457	106,175,002	IG C gene
Human	hg19	IGHA1			14	106,177,009	106,177,963	Sα region
Human	hg19	IGHG1	ENST00000390548.2	1-6	14	106,202,680	106,209,408	IG C gene
Human	hg19	IGHG1			14	106,210,947	106,211,836	Sγ region. Contains 7 breakpoints from 2 publications

Human	hg19	IGHG1			14	106,212,432	106,212,705	Sγ region. Contains 4 breakpoints from 2 publications
Human	hg19	IGHG3			14	106,213,054	106,213,828	nc region. Contains 3 breakpoints from 2 publications
Human	hg19	IGHG3	ENST00000390551.2	1-7	14	106,235,439	106,237,742	IG C gene
Human	hg19	IGHG3			14	106,239,969	106,240,335	Sγ region. Contains 6 breakpoints from 2 publications
Human	hg19	IGHM	ENST00000390559.2	1-4	14	106,320,349	106,322,323	IG C gene
Human	hg19	IGHM			14	106,324,109	106,324,904	Sμ region. Contains 7 breakpoints from 2 publications
Human	hg19	IGHM			14	106,325,023	106,325,998	Sμ region. Contains 12 breakpoints from 2 publications
Human	hg19	IGHM			14	106,326,019	106,326,648	Sμ region. Contains 18 breakpoints from 2 publications
Human	hg19	IGHM			14	106,326,933	106,327,173	Sμ region. Contains 3 breakpoints from 2 publications
Human	hg19	IGJ			14	106,328,547	106,329,405	nc region. Contains 8 breakpoints from 2 publications
Human	hg19	IGHJ6	ENST00000390560.2	1	14	106,329,406	106,329,470	IG J gene
Human	hg19	IGHJ6-J5			14	106,329,469	106,330,023	nc region. 11 breakpoints from 1 publication

Human	hg19	IGHJ5	ENST00000488476	1	14	106,330,024	106,330,072	IG J gene
Human	hg19	IGHJ5-J4			14	106,330,073	106,330,423	nc region
Human	hg19	IGHJ4	ENST00000461719	1	14	106,330,424	106,330,470	IG J gene
Human	hg19	IGHJ3	ENST00000463911	1	14	106,330,797	106,330,845	IG J gene
Human	hg19	IGHJ2	ENST00000390564.2	1	14	106,331,409	106,331,460	IG J gene
Human	hg19	IGHJ1	ENST00000390565.1	1	14	106,331,617	106,331,668	IG J gene
Human	hg19	IGHV1-2	ENST00000390594.2	1-2	14	106,452,671	106,453,170	IG V gene. Contains 3 breakpoints from 2 publications
Human	hg19	IGHV1-3	ENST00000390595.2	1-2	14	106,471,246	106,471,723	
Human	hg19	IGHV2-5	ENST00000390597	1-2	14	106,494,135	106,494,597	
Human	hg19	IGHV3-7	ENST00000390598	1-2	14	106,518,400	106,518,932	
Human	hg19	IGHV1-8	ENST00000390599.2	1-2	14	106,539,079	106,539,577	Associated predominantly with EBV-negative e/sBL
Human	hg19	IGHV1-18	ENST00000390605.2	1-2	14	106,641,563	106,642,056	3 breakpoint positions from 2 publications

Human	hg19	IGHV3-21	ENST00000390607.2	1-2	14	106,691,673	106,692,203	Associated with EBV-positive e/sBL
Human	hg19	IGHV3-23	ENST00000390609	1-2	14	106,725,198	106,725,733	2 breakpoint positions from 2 publications, more prevalent in DLBCL than s/eBL
Human	hg19	IGHV3-30	ENST00000390613.2	1-2	14	106,791,005	106,791,536	1 breakpoint position from 2 publications
Human	hg19	IGHV4-31	ENST00000438142.2	1-2	14	106,805,209	106,805,716	
Human	hg19	IGHV3-33	ENST00000390615.2	1-2	14	106,815,722	106,816,253	
Human	hg19	IGHV4-34	ENST00000390616.2	1-2	14	106,829,594	106,830,076	Equally prevalent in DLBCL
Human	hg19	IGHV4-39	ENST00000390619.2	1-2	14	106,877,619	106,878,126	
Human	hg19	IGHV1-46	ENST00000390622.2	1-2	14	106,967,049	106,967,788	2 breakpoints from 2 publications
Human	hg19	IGHV5-51	ENST00000390626.2	1-2	14	107,034,729	107,035,221	
Human	hg19	IGHV4-59	ENST00000455737.1	1-3	14	107,081,806	107,083,830	
Human	hg19	IGHV1-69	ENST00000390633.2	1-2	14	107,169,931	107,170,428	
Human	hg19	IGHV2-70	ENST00000390634.2	1-2	14	107,178,820	107,179,338	

Human	hg19	IGLJ1	ENST00000390320	1	22	23,235,872	23,235,998	
Human	hg19	IGLJ2	ENST00000390322.2	1	22	23,241,661	23,241,835	
Human	hg19	IGLJ3	ENST00000390324.2	1	22	23,247,030	23,247,205	
Human	hg19	IGLJ4	ENST00000390326.2	1	22	23,252,744	23,252,776	
Human	hg19	IGLJ5	ENST00000390327.2	1	22	23,256,408	23,256,479	
Human	hg19	IGLJ6	ENST00000390328	1	22	23,260,304	23,260,373	
Human	hg19	IGLV6-57	ENST00000390285.3	1-2	22	22,550,113	22,550,860	
Human	hg19	IGLV1-51	ENST00000390290.2	1-2	22	22,676,828	22,677,336	
Human	hg19	IGLV1-44	ENST00000390297.2	1-2	22	22,735,135	22,735,715	More frequent than that observed in DLBCL
Human	hg19	IGLV1-40	ENST00000390299.2	1-2	22	22,764,098	22,764,614	
Human	hg19	IGLV3-25	ENST00000390305.2	1-2	22	23,029,190	23,029,735	
Human	hg19	IGLV2-14	ENST00000390312.2	1-2	22	23,101,189	23,101,707	

Human	hg19	IGKJ1	ENST00000390242	1	2	89,161,398	89,161,435	
Human	hg19	IGKJ2	ENST00000390241.2	1	2	89,161,037	89,161,074	
Human	hg19	IGKJ3	ENST00000390240	1	2	89,160,733	89,160,770	
Human	hg19	IGKJ4	ENST00000390239.2	1	2	89,160,398	89,160,434	
Human	hg19	IGKJ5	ENST00000390238	1	2	89,160,080	89,160,117	
Human	hg19	IGKC	ENST00000390237.2	1	2	89,156,674	89,157,196	
Human	hg19	IGKV4-1	ENST00000390243.2	1-2	2	89,184,913	89,185,669	
Human	hg19	IGKV1-5	ENST00000496168.1	1-2	2	89,246,819	89,247,475	
Human	hg19	IGKV3-20	ENST00000492167.1	1-2	2	89,442,057	89,442,643	
Human	hg19	IGKV1-33	ENST00000473726.1	1-2	2	89,567,758	89,568,263	
Human	hg19	IGKV1-37	ENST00000465170.1	1-2	2	89,597,021	89,597,542	
Human	hg19	IGKV1-39	ENST00000498574.1	1-2	2	89,619,383	89,619,904	

Human	hg19	IGKV1-27	ENST00000498435	1-2	2	89,512,908	89,513,413	
Human	hg19	rs28372410			1	248,512,064	248,512,064	unique human snps
Human	hg19	rs4542826			2	33,413,949	33,413,949	unique human snps
Human	hg19	rs2717225			3	122,873,923	122,873,923	unique human snps
Human	hg19	rs2303808			5	39,074,296	39,074,296	unique human snps
Human	hg19	rs2286213			7	18,914,251	18,914,251	unique human snps
Human	hg19	rs7039358			9	90,401,638	90,401,638	unique human snps
Human	hg19	rs34084011			10	28,233,933	28,233,933	unique human snps
Human	hg19	rs7497712			15	100,821,647	100,821,647	unique human snps
Human	hg19	rs3816550			19	17,173,487	17,173,487	unique human snps
Human	hg19	rs723469			21	32,525,345	32,525,345	unique human snps

Table 5: Summary of Sequencing Metrics, n=308

Sequencing Metric (units)	Median (Range)
Coverage depth	1966 (97 – 5836)
Alignment (%)	2.82 (0.33 – 7.48)
Yield (gb)	5.7 (1.4 – 8.05)
Cluster (%)	88.56 (58.5 – 96.04)
Error rate (%)	0.56 (0.3 – 2.79)
Q30 (%)	93.59 (64.18 – 98.01)
Percentage panel covered at least 100x	98 (0 – 99)
Ti/tv ratio	1.44 (0 – 2.07)
Percentage of Reads Off-target	61.46 (37.91 – 99.98)

Table 6: Flagged Variants likely affected by Copy Number Alterations

Gene	Position	Change	VAF	ctDNA	Diagnosis	resid	flag
**TP53**	7,577,046	C/T	0.3905	825.0137	BL	0.2723072	Above expected (LOH-like)

**TP53**	7,577,539	G/A	0.3808	371.5303	BL	0.2843061	Above expected (LOH-like)
**TP53**	7,578,433	G/T	0.3756	570.4955	BL	0.2674448	Above expected (LOH-like)
**TP53**	7,578,445	A/G	0.3863	570.4955	BL	0.2781448	Above expected (LOH-like)
**MYC**	128,747,432	C/G	0.3837	4,587.44	BL	0.2187822	Above expected (LOH-like)
**MYC**	128,748,407	G/A	0.3968	2,467.74	BL	0.2487726	Above expected (LOH-like)
**MYC**	128,748,408	G/A	0.3975	2,467.74	BL	0.2494726	Above expected (LOH-like)
**MYC**	128,748,568	G/A	0.3446	570.4955	BL	0.2364448	Above expected (LOH-like)
**MYC**	128,748,621	C/G	0.393	825.0137	BL	0.2748072	Above expected (LOH-like)
**MYC**	128,748,676	T/C	0.3761	825.0137	BL	0.2579072	Above expected (LOH-like)
**MYC**	128,748,750	C/T	0.3897	4,587.44	BL	0.2247822	Above expected (LOH-like)
**MYC**	128,748,972	C/T	0.3527	592.7384	BL	0.2435044	Above expected (LOH-like)

**MYC**	128,749,107	-/C	0.369	2,446.02	non-BL	0.2212133	Above expected (LOH-like)
**MYC**	128,749,122	T/G	0.3779	825.0137	BL	0.2597072	Above expected (LOH-like)
**MYC**	128,749,252	G/C	0.3528	1,596.51	non-BL	0.2166334	Above expected (LOH-like)
**MYC**	128,749,255	C/T	0.3609	825.0137	BL	0.2427072	Above expected (LOH-like)
**MYC**	128,749,273	G/C	0.3938	825.0137	BL	0.2756072	Above expected (LOH-like)
**MYC**	128,749,344	T/A	0.3978	825.0137	BL	0.2796072	Above expected (LOH-like)
**MYC**	128,749,539	A/T	0.3983	742.3998	non-BL	0.2829787	Above expected (LOH-like)
**MYC**	128,749,927	C/T	0.3892	825.0137	BL	0.2710072	Above expected (LOH-like)
**MYC**	128,750,027	G/A	0.3837	3,607.07	BL	0.2253322	Above expected (LOH-like)
**MYC**	128,750,053	G/A	0.3833	2,197.77	BL	0.2384283	Above expected (LOH-like)
**MYC**	128,750,075	A/T	0.3878	3,607.07	BL	0.2294322	Above expected (LOH-like)

**MYC**	128,750,087	C/T	0.3767	3,607.07	BL	0.2183322	Above expected (LOH-like)
**MYC**	128,750,089	T/A	0.3968	4,587.44	BL	0.2318822	Above expected (LOH-like)
**MYC**	128,750,227	G/T	0.3176	388.5382	BL	0.2198896	Above expected (LOH-like)
**MYC**	128,750,297	A/C	0.325	388.5382	BL	0.2272896	Above expected (LOH-like)
**MYC**	128,750,396	G/C	0.3311	742.3998	non-BL	0.2157787	Above expected (LOH-like)

SUPPLEMENTARY DATA
