# Supplementary material for: Liquid biopsy for the diagnosis of EBV-positive Burkitt’s lymphoma in endemic areas
Source: Nat Med. 2026 Mar 19;32(5):1754–62. doi: 10.1038/s41591-026-04291-z (PMC13190244; doi:10.1038/s41591-026-04291-z)
Supplement: Supplementary file 2 — Reporting Summary [file 41591_2026_4291_MOESM2_ESM.pdf]

# nature portfolio

Corresponding author(s): Clara Chamba

Last updated by author(s): Jan 23, 2026

# Reporting Summary

Nature Portfolio wishes to improve the reproducibility of the work that we publish. This form provides structure for consistency and transparency in reporting. For further information on Nature Portfolio policies, see our Editorial Policies and the Editorial Policy Checklist.

## Statistics

For all statistical analyses, confirm that the following items are present in the figure legend, table legend, main text, or Methods section.

| n/a | Confirmed | |
|---|---|---|
| ☐ | ☒ | The exact sample size (*n*) for each experimental group/condition, given as a discrete number and unit of measurement |
| ☐ | ☒ | A statement on whether measurements were taken from distinct samples or whether the same sample was measured repeatedly |
| ☐ | ☒ | The statistical test(s) used AND whether they are one- or two-sided<br>*Only common tests should be described solely by name; describe more complex techniques in the Methods section.* |
| ☐ | ☒ | A description of all covariates tested |
| ☐ | ☒ | A description of any assumptions or corrections, such as tests of normality and adjustment for multiple comparisons |
| ☐ | ☒ | A full description of the statistical parameters including central tendency (e.g. means) or other basic estimates (e.g. regression coefficient) AND variation (e.g. standard deviation) or associated estimates of uncertainty (e.g. confidence intervals) |
| ☐ | ☒ | For null hypothesis testing, the test statistic (e.g. *F*, *t*, *r*) with confidence intervals, effect sizes, degrees of freedom and *P* value noted<br>*Give P values as exact values whenever suitable.* |
| ☒ | ☐ | For Bayesian analysis, information on the choice of priors and Markov chain Monte Carlo settings |
| ☒ | ☐ | For hierarchical and complex designs, identification of the appropriate level for tests and full reporting of outcomes |
| ☐ | ☒ | Estimates of effect sizes (e.g. Cohen's *d*, Pearson's *r*), indicating how they were calculated |

*Our web collection on statistics for biologists contains articles on many of the points above.*

## Software and code

Policy information about availability of computer code

| | |
|---|---|
| Data collection | Clinical and laboratory data were collected using electronic data capture with REDCap (version 13.1.0, © 2025 Vanderbilt University), hosted at Muhimbili National Hospital. Data were entered manually at the point of care using a tablet device. No commercial or custom code was used for data collection. cfDNA sequence data was collected in FASTQ format and stored by a bespoke pipeline in a HIPPAA compliant, AWS cloud. |
| Data analysis | Clinical and laboratory data analysis was performed in R version 4.2.3 using open-source packages. Sequence data was analsyed in a bespoke pipeline. Analysis incorporated both custom tools (udini, elduderino, trim) and widely used open-source bioinformatics software (Samtools, BWA-MEM2, Varscan, VEP, IgCaller, GRIDSS) All codes used in the analysis are accessible at https://github.com/ClaraClaudius/CLINICAL-VALIDATION-OF-LIQUID-BIOPSY-FOR-FASTER-DIAGNOSIS-OF-EBV-POSITIVE-BURKITT-LYMPHOMA.git. Tools used for the bioinformatics analysis are as follows:<br>1. Udini - This is a locally created tool. It extracts UMIs from FASTQ files. It combines all of the reads for a sample into one interleaved FASTQ. It also puts QC information into the stats.json file, specifically,total reads and reads that are invalid for being too short (threshold is 50bp)<br>2. BWA-MEM2 (version 2.2.1) - This is an open-source reimplementation of BWA-MEM, was used for aligning sequencing reads to the GRCh37 reference genome<br>3. Elduderino - This is a locally created tool. It is used to de-duplicate the read. Read families are collapsed into consensus reads. Elduderino also adds smetrics to the stats.json file, including, error rates, and family sizes<br>4. size - Locally developed script. Uses the read pairs to calculate fragment sizes and saves fragment sizes to stats.json and plotted in the sizes.pdf file<br>5. ontarget - locally created tool. This takes the deduplicated, and re-aligned, SAM file along with a bed file as it's input; If any of the read overlaps with any region of interest in the bed file then that read is kept. This is also were levels of EBER1, EBER2 and EBNA-2 are calculated. The mean depth of coverage for each EBV gene is divided by the mean depth for all other genes to give an approximation of the number of |

copies present relative to other genes. Also adds off target rate to stats.json file
6. trim - Trims the last base from the end of each read improving the quality of variant calling
7. covermi_stats - This is a locally developed python script, which uses the locally developed CoverMi package. It takes BAM and panel folder as input. The panel folder contains the bed file as well as some information about which transcripts to use and exon locations. Generates coverage statistic and calculates the mean depth across all targets as well as the percentage of targets that were covered at 30, 100, 500, 1000 and 2000x. It also gives this statistics on a per gene basis
8. call_variants - locally developed python script.This is essentially a wrapper script that is used to easily call multiple different variant callers as well as annotating the resulting VCF files with ensembls variant effect predictor (VEP). Has many inputs including the BAM file, reference genome, variant caller to use (current options are vardict, varscan and mutect2) along with the minimum VAF and minimum number of alternate reads required to call a variant
9. vcf_stats - custom python script - This provides some statistics about the VCFs including total number of variants, total number of insertions and deletions, or InDels, the transition to transversion or ti/tv ratio. All of this metrics get added to the stats.json file
10. IgCaller (Version 1.2 software utilizing the hg19 reference genome)- Python script designed to fully characterize the immunoglobulin gene rearrangements and oncogenic translocations in lymphoid neoplasms.
11. GRIDSS (the Genomic Rearrangement IDentification Software Suite) version 2.13.2 - structural variant caller

For manuscripts utilizing custom algorithms or software that are central to the research but not yet described in published literature, software must be made available to editors and reviewers. We strongly encourage code deposition in a community repository (e.g. GitHub). See the Nature Portfolio guidelines for submitting code & software for further information.

# Data

Policy information about availability of data

All manuscripts must include a data availability statement. This statement should provide the following information, where applicable:

- Accession codes, unique identifiers, or web links for publicly available datasets
- A description of any restrictions on data availability
- For clinical datasets or third party data, please ensure that the statement adheres to our policy

The raw sequencing data and individual-level clinical data generated in this study cannot be made publicly available owing to ethical and data protection restrictions, as they include information from human participants collected under institutional approvals that do not permit open public deposition. Data access is governed by the study consortium and collaborating centers in Tanzania and Uganda. Requests to access the underlying anonymized data will be reviewed by the consortium's data access committee in consultation with all participating institutions.
If a request is deemed scientifically sound and compliant with applicable institutional, national, and international data protection regulations, de-identified and anonymized data will be shared following the execution of a data transfer agreement. Processed and anonymized data supporting the findings of this study are available in the GitHub repository https://github.com/ClaraClaudius/CLINICAL-VALIDATION-OF-LIQUID-BIOPSY-FOR-FASTER-DIAGNOSIS-OF-EBV-POSITIVE-BURKITT-LYMPHOMA.git. This repository constitutes the source data for this paper and is provided to ensure transparency and reproducibility. All data-sharing requests should be addressed to the corresponding author (clarachamba@gmail.com). Timelines for review, approval, and data transfer may vary depending on the scope and regulatory requirements of each request

# Research involving human participants, their data, or biological material

Policy information about studies with human participants or human data. See also policy information about sex, gender (identity/presentation), and sexual orientation and race, ethnicity and racism.

| | |
|---|---|
| Reporting on sex and gender | Sex (a biological attribute) was recorded for all participants based on clinical records. Gender identity was not assessed. The study included both male and female participants. Sex was evaluated in a univariate analysis using the chi-squared test to assess its association with Burkitt lymphoma diagnosis, and was also included as a covariate in the multivariable diagnostic models. The results of these analyses are reported in the main text and source data. Consent for sharing de-identified sex-disaggregated data was not obtained; therefore, only aggregate results are reported |
| Reporting on race, ethnicity, or other socially relevant groupings | The only socially relevant categorization variable used in this study was sex, recorded as male or female based on clinical records. Gender identity, race, ethnicity, and socioeconomic status were not collected or analyzed. Sex was included as a variable to assess potential biological associations with diagnosis and was incorporated into both univariate and multivariable models. No proxy variables were used in place of other social constructs. Confounding was addressed by including clinically relevant covariates in multivariable logistic regression models, including age, LDH, EBV status, MYC-related molecular features, and cfDNA levels |
| Population characteristics | Covariate-relevant characteristics of the study participants included clinical and molecular variables collected to assess their diagnostic relevance and association with Burkitt lymphoma (BL). Clinical variables included age, sex, LDH level, duration of symptoms, and presence of jaw or abdominal mass. Molecular variables incorporated into the analysis included: median variant allele frequency (VAF) of somatic mutations in MYC, TP53, and ID3; circulating tumor DNA (ctDNA), calculated as the product of cfDNA concentration and median VAF; the absolute number of MYC intron 1 and exon 2 mutations; and EBV-related features including EBER1, EBER2, and EBNA2 copy number (copies per cell), the maximum value of EBV copies per cell (EBVmax), EBV fragment size ratio (EBVSR), EBV DNA proportion (EBVP), and entropy measures for EBV and autosomal DNA fragments. These variables were derived from targeted next-generation sequencing data and used as covariates in univariate and multivariable models to identify predictors of BL diagnosis |
| Recruitment | Participants were recruited prospectively from four referral hospitals in Tanzania and Uganda (Muhimbili National Hospital, Kilimanjaro Christian Medical Centre, Bugando Medical Centre, and St. Mary's Hospital Lacor) between August 2019 and July |

2023. Inclusion was limited to children and young adults aged 3–25 years with a clinical suspicion of lymphoma who provided informed consent. Patients who had previously received chemotherapy, immunotherapy, investigational agents, or participated in clinical trials were excluded. As recruitment was limited to referred cases presenting to tertiary centers, there is a potential for referral bias, which may overrepresent patients with more advanced or complex disease presentations. Self-selection bias is unlikely, as patients were enrolled consecutively based on clinical eligibility and willingness to consent. However, the exclusion of pretreated cases may limit generalizability to treatment-naive populations

| | |
|---|---|
| Ethics oversight | This study was approved by the Oxford Tropical Research Ethics Committee (OxTREC: 15-19), the National Institute of Medical Research (NIMR) in Tanzania (NIMR/HQ/R.8a/Vol.IX/3408), and the Uganda National Council of Science and Technology (UNCST: HS529ES) |

Note that full information on the approval of the study protocol must also be provided in the manuscript.

# Field-specific reporting

Please select the one below that is the best fit for your research. If you are not sure, read the appropriate sections before making your selection.

☒ Life sciences ☐ Behavioural & social sciences ☐ Ecological, evolutionary & environmental sciences

For a reference copy of the document with all sections, see nature.com/documents/nr-reporting-summary-flat.pdf

# Life sciences study design

All studies must disclose on these points even when the disclosure is negative.

| | |
|---|---|
| Sample size | A formal sample size calculation was conducted to ensure sufficient power for evaluating diagnostic performance. Using the binomial proportion formula with Z=1.96 (95% confidence), p=0.8 (expected sensitivity), and d=0.10 (margin of error), the minimum number of positive cases required was estimated to be 62, corresponding to a total sample size of approximately 124 participants (assuming a 1:1 case-to-control ratio). Additionally, using the events-per-variable (EPV) method with a conservative threshold of 20 and up to 18 predictors, we estimated a total required sample size of 720 participants. However, due to the prospective nature of the study and unforeseen disruptions during the COVID-19 pandemic—including delays in clinical workflows and research staffing—we were unable to reach this target. The final cohort included 212 participants, of whom 81 were diagnosed with Burkitt lymphoma. To address this limitation, we employed LASSO (Least Absolute Shrinkage and Selection Operator) regression, which performs regularization and variable selection, thereby reducing over fitting in models with relatively small sample sizes and many candidate predictors. We also used 10-fold cross-validation to internally validate model performance and assess generalizability. These strategies align with best practices for model development and transparent reporting in constrained-sample settings, as recommended by the TRIPOD guidelines. |
| Data exclusions | Participants with missing outcome data were excluded from the analysis. This exclusion was predefined to maintain the integrity of outcome-based comparisons. Only individuals lacking key clinical endpoints or follow-up information necessary for primary analysis were removed prior to statistical evaluation. For other missing data, such as covariates, appropriate imputation methods were applied to minimize bias and retain statistical power. |
| Replication | We took several measures to ensure the reproducibility of our findings. All sequencing and data processing were performed using standardized protocols and quality control criteria applied uniformly across samples. Bioinformatic analyses were conducted using version-controlled pipelines and documented custom scripts, with core steps (e.g., alignment, variant calling, EBV quantification) implemented through reproducible workflows.For model development and evaluation, we used 10-fold cross-validation to verify internal reproducibility and reduce overfitting. All statistical analyses were scripted in R and Python, and the full codebase is publicly available along with the source data used to generate the results.All attempts to replicate the main findings across cross-validation folds and within subgroups were successful and consistent. There were no results in this study that could not be reproduced. |
| Randomization | This was a prospective observational study. Participants were not randomly allocated into experimental groups. Instead, diagnostic groupings (e.g., Burkitt lymphoma vs. non-Burkitt lymphoma) were based on final clinical and pathological diagnoses. As such, group assignment reflected natural clinical presentation rather than study-driven allocation. To control for potential confounding, relevant clinical and molecular covariates (e.g., age, LDH, EBV status) were included in multivariable regression models and penalized regression techniques (LASSO) were applied during model development. |
| Blinding | Blinding of investigators was not applicable during participant recruitment or diagnostic group assignment, as group status was determined retrospectively based on clinical and pathological findings. However, data analysis—including cfDNA quantification, variant calling, and model development—was performed using automated pipelines and pre-specified statistical workflows, reducing the potential for bias. Investigators conducting statistical analysis were aware of group labels to enable supervised model training. Given the objective nature of the molecular measurements and algorithm-driven methods used, the absence of blinding was not expected to influence study outcomes. |

# Reporting for specific materials, systems and methods

We require information from authors about some types of materials, experimental systems and methods used in many studies. Here, indicate whether each material, system or method listed is relevant to your study. If you are not sure if a list item applies to your research, read the appropriate section before selecting a response.

## Materials & experimental systems

| n/a | Involved in the study |
|---|---|
| ☒ | ☐ Antibodies |
| ☒ | ☐ Eukaryotic cell lines |
| ☒ | ☐ Palaeontology and archaeology |
| ☒ | ☐ Animals and other organisms |
| ☒ | ☐ Clinical data |
| ☒ | ☐ Dual use research of concern |
| ☒ | ☐ Plants |

## Methods

| n/a | Involved in the study |
|---|---|
| ☒ | ☐ ChIP-seq |
| ☒ | ☐ Flow cytometry |
| ☒ | ☐ MRI-based neuroimaging |

# Plants

| | |
|---|---|
| Seed stocks | *Report on the source of all seed stocks or other plant material used. If applicable, state the seed stock centre and catalogue number. If plant specimens were collected from the field, describe the collection location, date and sampling procedures.* |
| Novel plant genotypes | *Describe the methods by which all novel plant genotypes were produced. This includes those generated by transgenic approaches, gene editing, chemical/radiation-based mutagenesis and hybridization. For transgenic lines, describe the transformation method, the number of independent lines analyzed and the generation upon which experiments were performed. For gene-edited lines, describe the editor used, the endogenous sequence targeted for editing, the targeting guide RNA sequence (if applicable) and how the editor was applied.* |
| Authentication | *Describe any authentication procedures for each seed stock used or novel genotype generated. Describe any experiments used to assess the effect of a mutation and, where applicable, how potential secondary effects (e.g. second site T-DNA insertions, mosiacism, off-target gene editing) were examined.* |
